# Effectiveness and Safety of Colorectal Cancer Screening Via Fecal Occult Blood Testing: A Systematic Review and Meta-Analysis

**DOI:** 10.1007/s12029-026-01605-9

**Published:** 2026-09-23

**Authors:** Mayra Calil Jorge Frankenfeld, Giovanna Camarotto Patah, Luiza Teixeira Soares, Sarah Nascimento Silva, Fernando Gomes Romeiro, Vania dos Santos Nunes Nogueira

**Affiliations:** 1 Faculdade de Medicina, Universidade Estadual Paulista (UNESP), Botucatu, Sao Paulo, Brazil; 2 Faculty of Medicine of Jundiaí (FMJ), Jundiaí, São Paulo, Brazil; 3 René Rachou Institute, Oswaldo Cruz Foundation, FIOCRUZ, Belo Horizonte, Minas Gerais Brazil

**Keywords:** Colorectal cancer, Population screening, Fecal occult blood test, Randomized clinical trial, Mortality

## Abstract

**Purpose:**

This systematic review evaluated the effectiveness of population-based colorectal cancer (CRC) screening programs using fecal occult blood tests (gFOBT or FIT) among adults aged 45–75 years.

**Methods:**

We included studies comparing participants in population-based CRC screening programs with unscreened individuals. Outcomes were all-cause mortality, CRC-specific mortality, CRC incidence, and stage IV CRC incidence. Two reviewers independently performed study selection, data extraction, and risk-of-bias assessment. Pooled RRs were calculated by meta-analysis, and certainty of evidence was assessed using GRADE.

**Results:**

No difference was observed in all-cause mortality (RR = 1.00, 95% CI 0.99–1.01; 5 studies, 1,474,576 participants; moderate-certainty evidence). RCTs evaluating gFOBT demonstrated a reduction in CRC-specific mortality (RR = 0.89, 95% CI 0.84–0.94; 5 studies, 1,474,576 participants; moderate-certainty evidence), corresponding to 38 fewer CRC deaths per 100,000 individuals screened (95% CI, 55 fewer to 21 fewer). Non-RCTs evaluating FIT reported larger reductions in CRC-specific mortality (RR = 0.21, 95% CI 0.20–0.21; 5 studies, 7,435,956 participants), although the certainty of evidence was low. RCTs showed no significant effect on CRC incidence or stage IV CRC incidence, whereas non-RCTs evaluating FIT suggested reductions in both outcomes; however, the certainty of evidence was also low.

**Conclusion:**

Fecal occult blood test–based screening reduces CRC-specific mortality but not all-cause mortality. FIT-based screening may provide additional benefits by reducing CRC incidence and stage IV disease; however, these findings are supported by low-certainty evidence and require confirmation in RCTs.

**Supplementary Information:**

The online version contains supplementary material available at https://doi.org/10.1007/s12029-026-01605-9.

## Introduction

Colorectal cancer (CRC) is the third most commonly diagnosed cancer worldwide, accounting for approximately 10% of all cancer cases, and the second leading cause of cancer-related death globally [1]. In 2022, an estimated 1.9 million new cases of CRC and more than 900,000 deaths occurred worldwide, highlighting its substantial global burden. It predominantly affects individuals aged ≥ 50 years, although incidence is increasing among younger adults [1].

CRC is particularly amenable to screening because it commonly develops through a prolonged preclinical phase, often progressing from adenomatous polyps and other precursor lesions to invasive cancer [2]. Detection and removal of premalignant lesions can prevent CRC, while diagnosis at an earlier stage increases the likelihood of curative treatment and reduces morbidity and mortality [3]. The lower the stage is, the greater the likelihood of curative treatment. In contrast, Stage IV colorectal cancer is associated with a 5-year survival rate of approximately 13–18% [4].

Screening for CRC can be performed using several methods, including colonoscopy, flexible sigmoidoscopy, CT colonography, stool DNA testing, and fecal occult blood testing [5]. Among these, colonoscopy is considered the reference standard and can be used as both a screening and diagnostic procedure, whereas positive results from noninvasive tests generally require confirmation by colonoscopy. Colonoscopy has excellent accuracy, with 95% sensitivity and 98% specificity; however, it is difficult to implement in countries with continental dimensions, a large population, and a developing economy [5]. In contrast, fecal occult blood testing is inexpensive, widely accessible, noninvasive, and more feasible for large-scale population-based screening programs.

There are two methods available to search for fecal occult blood: guaiac (gFOBT) and the immunochemical method (FIT) [6]. The first is a colorimetric method, which is based on the oxidation of guaiac catalyzed by the action of peroxidase present in hemoglobin, resulting in a blue color. FIT uses specific antibodies against human hemoglobin or other blood components. The main advantages of FIT over the gFOBT method include the fact that there is no need for preparation before the exam (food and medication restrictions); it is not sensitive to bleeding originating from the upper gastrointestinal tract (because it only reacts with undegraded globin); and it has a better detection limit of hemoglobin per gram of feces [5].

Randomized trials of stool-based CRC screening have predominantly evaluated gFOBT and have established a reduction in CRC-specific mortality [7–9]. However, FIT has largely replaced gFOBT in contemporary screening programs because of its practical and analytical advantages, while evidence regarding its effect on mortality derives predominantly from non-randomized population-based studies [10–12]. Consequently, the evidence supporting stool-based screening currently comprises two distinct but complementary bodies of evidence: randomized evidence establishing the mortality benefit of gFOBT-based screening and more recent observational evidence evaluating the effectiveness of FIT in contemporary practice.

Therefore, this systematic review aimed to provide an updated and comprehensive assessment of the effects of population-based CRC screening programs using fecal occult blood tests (gFOBT or FIT) among adults aged 45–75 years.

## Methods

This systematic review was performed according to the recommendations of the Cochrane Collaboration [13] and reported according to the PRISMA (Preferred Reporting Items for Systematic Reviews and Meta-Analysis) Statement [14]. Its protocol was registered on the International Prospective Register of Systematic Reviews (PROSPERO) - Registration number CRD42020169541.

### Eligibility Criteria

For this systematic review, randomized controlled studies (RCTs) in which patients were randomly allocated into two groups (with and without intervention) were included. Owing to the lack of RCTs that evaluated mortality outcomes with screening via the FIT method, prospective population cohort studies that used the FIT method and evaluated mortality were included. The included studies followed the “PICO” structure described below.

### Participants (P)

Participants were defined as adult individuals aged between 45 and 75 years, with a standard risk for CRC, who were asymptomatic for this cancer, and who underwent population screening for this neoplasm. The term “standard risk” applies to people who are not predisposed to CRC.

### Types of Interventions (I)

Individuals in the intervention group underwent fecal occult blood tests via gFOBT or FIT.

### Comparison (C)

Individuals in the control group were defined as those who did not participate in population screening for CRC.

### Outcomes (O)

All-cause mortality and CRC-specific mortality were considered main outcomes, and the other outcomes included the incidence of CRC and its incidence at stage IV (distant metastases), adverse events related to colonoscopy with and without polypectomy (bleeding or intestinal perforation), quality of life, survival within five years and the need for chemotherapy and radiotherapy.

### Exclusion Criteria

We excluded studies whose screening method was not gFOBT or FIT, studies whose population corresponded to symptomatic patients or individuals at high risk for CRC development, non-population-based screening studies, studies that did not assess mortality as an outcome, and non-RCTs evaluating gFOBT.

### Identification of Studies - Electronic Databases

A comprehensive literature search was conducted in MEDLINE (via PubMed), Embase, LILACS, and the Cochrane Central Register of Controlled Trials (CENTRAL). The search strategy combined controlled vocabulary terms (e.g., MeSH and Emtree terms) and free-text keywords related to colorectal cancer, colorectal neoplasms, fecal occult blood testing, guaiac fecal occult blood testing, colorectal cancer screening, and cohort studies. Operators (“AND” and “OR”) were used to combine search terms as appropriate. For MEDLINE and Embase, validated search filters recommended by the Cochrane Collaboration for identifying RCTs were applied. Additionally, MeSH and Emtree terms related to cohort studies were incorporated into the search strategy to capture eligible observational studies. No restrictions were imposed on language, publication date, or publication status. The initial search was performed on October 18, 2019, and updated on April 19, 2024. Detailed search strategies for all databases are provided in Supplementary Material.

EndNote X9 citation management software was used to download references and remove duplicate entries. The initial screening of abstracts and titles was performed via the free web application Rayyan QCRI [15].

### Study Selection

Titles and abstracts were independently screened in duplicate by pairs of reviewers to determine potential eligibility. Three reviewers (LTS, SNS and VSNN) screened studies published up to 2019, while two reviewers (MCJF and GCP) screened studies published between 2019 and 2024. Studies selected for full-text review were subsequently assessed for consistency with the predefined PICO framework. Disagreements were resolved through discussion, with consensus reached among the reviewers.

### Data Extraction and Management

The three reviewers (LTS, MCJF and GCP) used a standard form to extract the following data from the selected studies: year of publication, country, randomized or non-RCT, screening frequency, type of intervention and comparison, number of patients randomized to the intervention and comparison groups, main outcomes and mean age of participants.

To ensure consistency between reviewers, we performed a calibration exercise before beginning the review. In the case of duplicate publications or multiple reports from the primary study, data extraction was optimized using the best information available for all items in the same study.

### Assessment of Risk of Bias in Included Studies

Risk of bias was assessed using the revised Cochrane Risk of Bias tool (RoB 2) [16], which evaluates five domains: randomization, deviations from intended interventions, missing outcome data, outcome measurement, and selective reporting. Each domain was independently assessed by two reviewers as having low risk of bias, some concerns, or high risk of bias.

For non-RCTs, risk of bias was assessed using the ROBINS-I tool [17], which evaluates seven domains: confounding, participant selection, intervention classification, deviations from intended interventions, missing data, outcome measurement, and selective reporting. Each domain was rated as low, moderate, serious, or critical risk of bias, and an overall risk-of-bias judgment was subsequently assigned.

### Data Analysis

Meta-analyses were conducted using Stata Statistical Software, version 16 (Stata Corp LLC, College Station, TX, USA). The primary effect measures were the relative risks (RRs) of colorectal cancer incidence, colorectal cancer mortality, all-cause mortality, and adverse events, with corresponding 95% confidence intervals (CIs). Pooled effect estimates were calculated using the DerSimonian–Laird random-effects model to account for potential clinical and methodological heterogeneity across studies.

### Subgroup Analysis

The studies were divided according to their design into RCT or non-RCT studies. The meta-analyses were performed separately for each subgroup.

### Sensitivity Analysis

A leave-one-out sensitivity analysis was performed by sequentially excluding each study from the meta-analysis and recalculating the pooled effect estimate. Additionally, sensitivity analyses using a fixed-effects model were conducted, and the corresponding sensitivity analysis plots were generated to evaluate the robustness and stability of the findings.

### Heterogeneity Analysis

Inconsistencies between the results of the studies included were ascertained by visual inspection of forest plots (no overlap of CIs around the effect estimates of the individual studies) and by Higgins or I^2^ statistics, in which I^2^ > 50% indicated a moderate probability of heterogeneity, and by chi-square tests (Chi2), where *p* < 0.10 indicated heterogeneity [13].

### Publication Bias

Publication bias was planned to be assessed when at least 10 studies were available for a given meta-analysis, as recommended by methodological guidelines [13]. In such cases, funnel plot asymmetry would be visually inspected and formally evaluated using Egger’s regression test.

### Quality of Evidence

The quality of the evidence of the effect size was assessed according to the Grading of Recommendations Assessment, Development, and Evaluation (GRADE) guidelines [18].

## Results

**Fig. 1:**
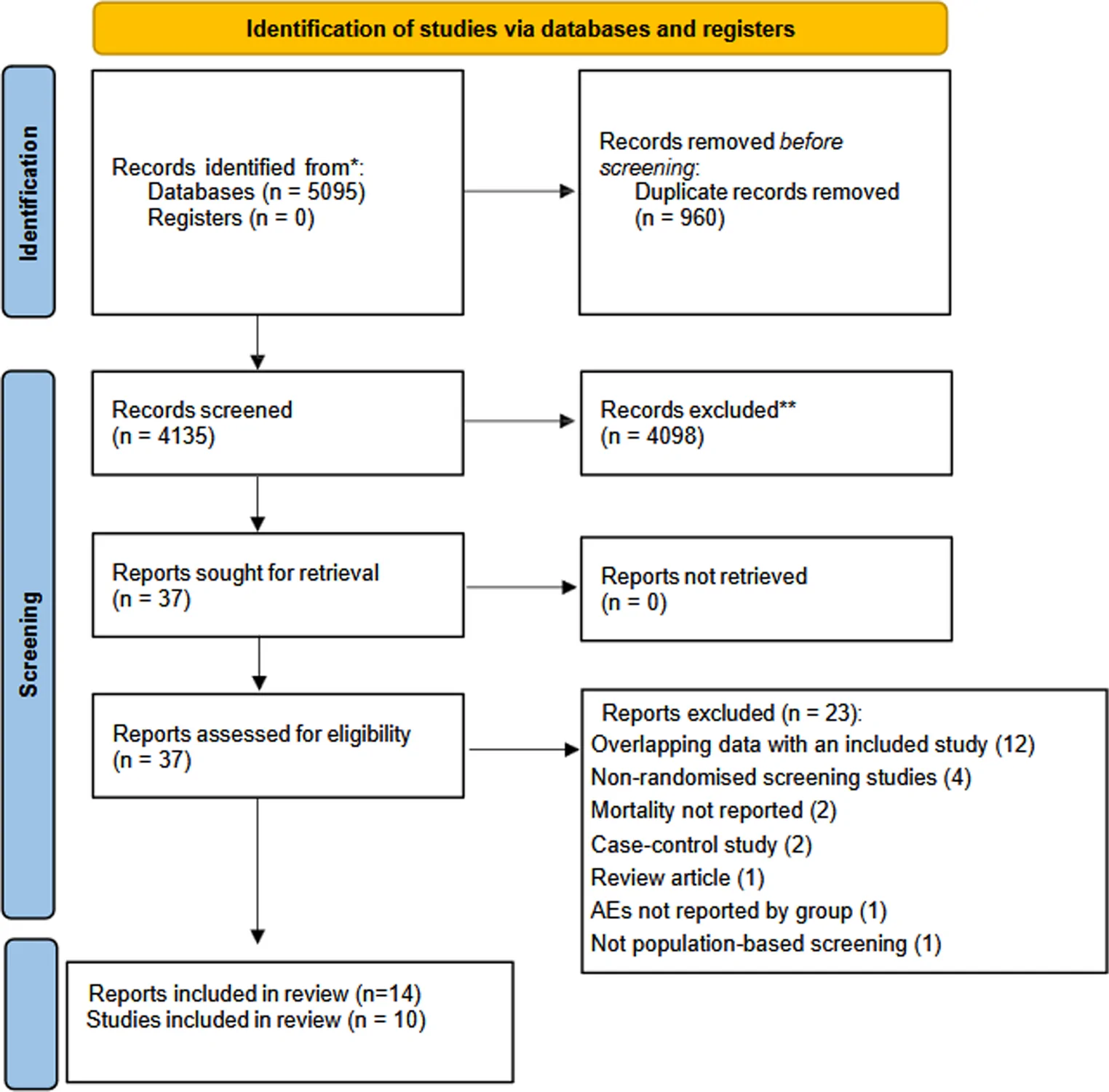
PRISMA flow diagram of the study selection process

The search strategy identified 4,135 records after duplicate removal. Of these, 37 studies were selected for full-text review (Fig. 1). Ultimately, 10 studies met the eligibility criteria and were included in the review, comprising a total of 14 publications, as some studies were reported in more than one publication [7–12, 19–26].

A total of 23 studies were excluded for the following reasons: overlapping data with an included study (*n* = 12); non-RCT screening (*n* = 4); no mortality data reported (*n* = 2); case–control design (*n* = 2); review article without primary data (*n* = 1); adverse events not reported by study group (*n* = 1); and non-population-based screening (*n* = 1). The list of excluded studies and their corresponding references are provided in the Supplementary Material.

### Study Characteristics

Among the 10 studies included, five were RCTs that used gFOBT as an intervention and no screening as a comparator. Most studies applied gFOBT with biennial frequency, and only one study used different screening intervals (intervention group participants were invited to the first screening round and to one or two rescreening rounds) [22]. The five remaining studies were prospective cohort studies evaluating FIT-based screening compared with no screening. FIT was predominantly performed biennially, with screening periods ranging from approximately 4 to 11 years, reflecting repeated screening over time; one study evaluated a single screening round.

CRC screening was initiated between 45 and 60 years of age and generally continued until 69 to 80 years of age. The main characteristics of all included studies, including screening frequency and duration of the screening period, are summarized in Table 1.

**Table 1:** Characteristics of the included studies

Author	Year	Country	Design	Intervention	Frequency	Nº Participants CRC screening/control	Age at screening (years)	Screening Time	Follow-up	Outcomes
Baldacchini	2022	Italy	Cohort	FIT	Biennial	380,651/ 327,100	50–69	2005–2016	11 years	All-M, CRC -M
Blom	2024	Sweden	Cohort	FIT, FOBT	Biennial	203,670/ 175,778	60–69	2008–2012	10.8 years	CRC -M
Chiu	2015/2021	Taiwan	Cohort	FIT	Biennial	3,067,853/ 2,349, 846	50–69	2004–2009	10 years	CRC-M. CRC -F, CRC-IV
Kronborg	2004	Denmark	RCT	gFOBT	Biennial	431,190/ 430,755	45–75	1985–2002	17 years	All-M, CRC-M, CRC -F, CRC -IV
Lindholm	2008	Sweden	RCT	gFOBT	Twice	34,144/ 34,164	60–64	1982–1995	15.5 years	All-M, CRC -M, CRC -F
Shaukat/ Mandel	2000	EUA	RCT	gFOBT	Biennial	15,587/ 15,394	60–80	1976-82/86–92	18/30 years	All-M, CRC -M, CRC -F, CRC-IV
Njor	2022	Denmark	Quasi- experimental	FIT	Once	262,893/ 634,919	50–71	2014–2017	3.3 Years	CRC -M
Pitkaniemi/Paimela Koskenvuo	2015/2019/2010	Finland	RCT	gFOBT	Biennial	180,210/ V	60–69	2004–2012	4.5 years	All-M, CRC -M, CRC -F, CRC -IV
Scholefield	2012	United Kingdom	RCT	gFOBT	Biennial	76,466/ 76,384	45–74	1981–1991	20 years	All-M, CRC -M, CRC-F, CRC-IV
Ventura	2014	Italy	Cohort	FIT	Biennial	6,961/ 26,285	50–70	1993–1999	11 years	CRC-M. CRC -F

*FIT*: fecal immunochemical test; *gFOBT*: guaiac-based fecal occult blood test; *RCT*: randomized controlled trial; *CRC*: colorectal cancer; *CRC-M*: colorectal cancer–specific mortality; *CRC-F*: frequency (incidence) of colorectal cancer; *CRC-IV*: frequency of stage IV colorectal cancer; *All-M*: all-cause mortality

### Risk of Bias

**Fig. 2:**
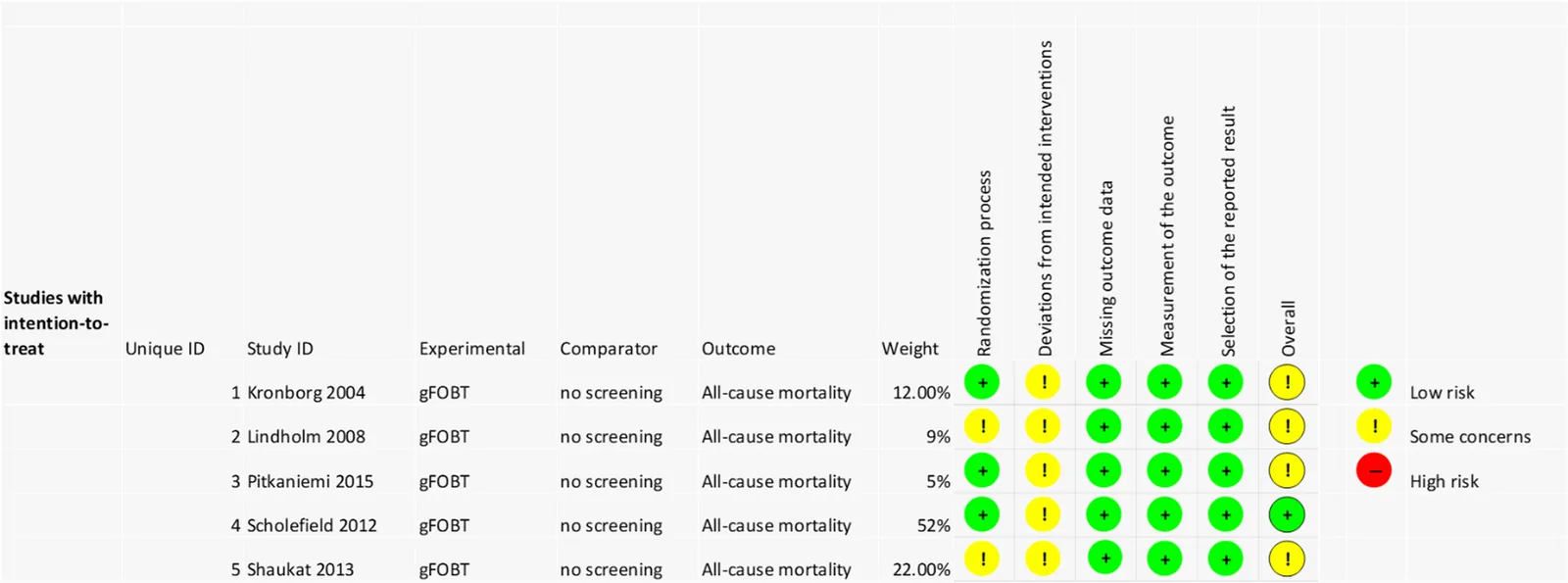
Risk of bias assessment of the included randomized controlled trials according to the RoB 2 tool

No RCT was judged to be at high risk of bias in any domain of the RoB 2 tool. Overall, most randomized trials were assessed as having low risk of bias or some concerns across the evaluated domains, and their detailed assessments are presented in Fig. 2. According to the ROBINS-I tool, four non-RCTs were judged to be at serious risk of bias and one at moderate risk of bias, with detailed evaluations provided in the Supplementary Material. The main concerns involved residual confounding, self-selection into screening participation, healthy-volunteer bias, and contamination between comparison groups. In contrast, the risk of bias related to outcome measurement, missing data, and selective reporting was generally low across studies.

### Meta-Analysis

**Fig. 3:**
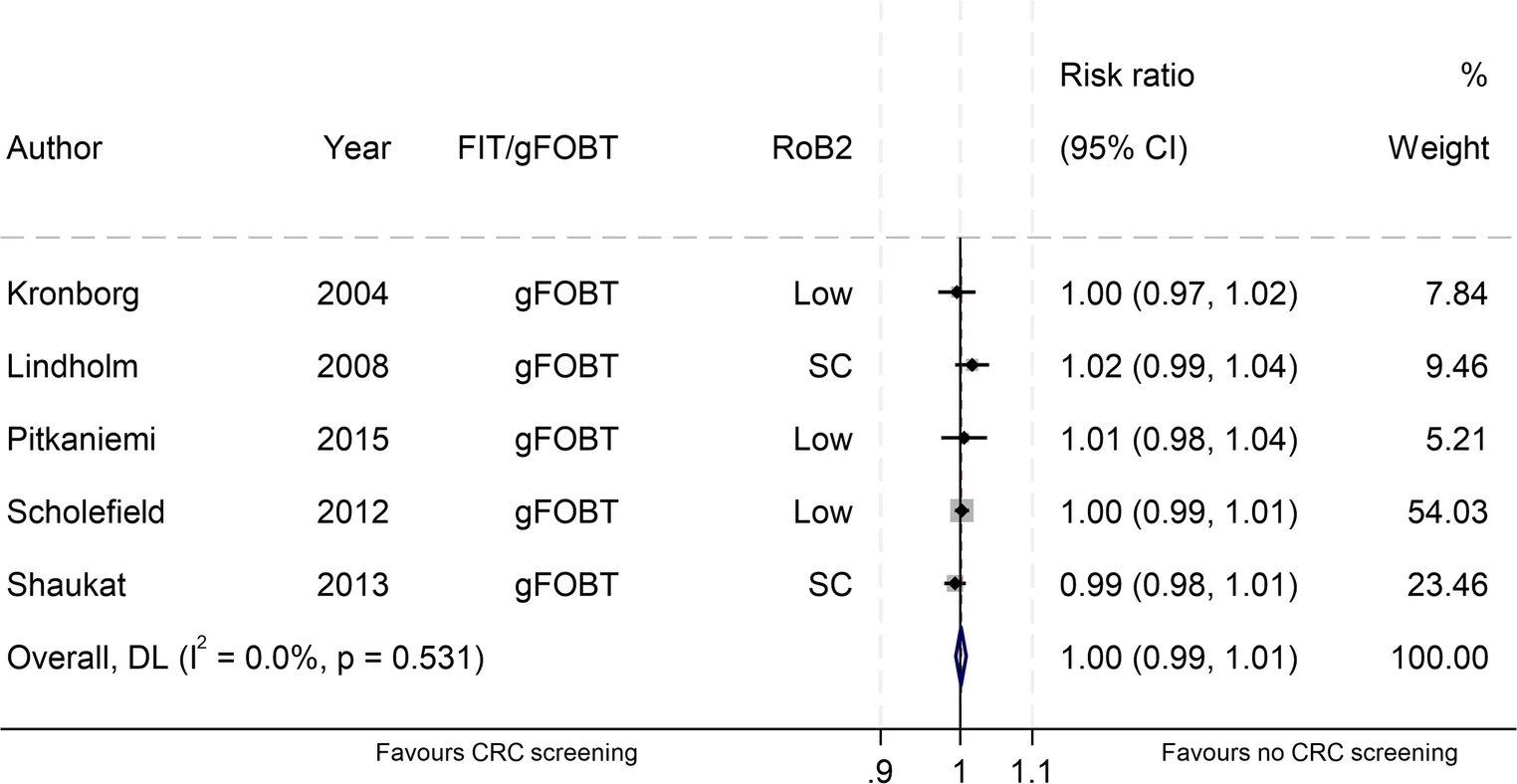
Forest plot of the effect of colorectal cancer screening on all-cause mortality compared with no screening. Low = low risk of bias; SC = some concerns, according to the Cochrane Risk of Bias 2 (RoB 2) tool. Note: Weights are from random-effects model

Regarding all-cause mortality, there was no evidence that CRC screening reduced mortality risk (RR: 1.0, 95% CI: 0.99–1.01; 5 RCTs, 1,474,576 participants; moderate certainty of evidence; Fig. 3; Table 2).

**Table 2:** Summary of findings for colorectal cancer screening with fecal occult blood testing and certainty of evidence assessed using GRADE

Certainty assessment							№ of patients		Effect		Certainty	Importance
№ of studies	Study design	Risk of bias	Inconsistency	Indirectness	Imprecision	Other considerations	Colorectal Cancer Screening Using gFOBT	No screening	Relative (95% CI)	Absolute (95% CI)		
All-cause Mortality (follow-up: range 23 months to 30 years; assessed with: gFOBT)												
5	randomized trials	serious^a^	not serious	not serious	not serious	none	82,481/737,597 (11.2%)	82,137/736,979 (11.1%)	**RR 1.00** (0.99 to 1.01)	**0 fewer per 100.000** (from 111 fewer to 111 more)	⨁⨁⨁◯ Moderate	CRITICAL
**Cancer Colorrectal Mortality (follow-up: range 23 months to 30 years; assessed with: gFOBT)**												
5	randomized trials	serious^a^	not serious	not serious	not serious	none	2262/737,597 (0.3%)	2538/736,979 (0.3%)	**RR 0.89** (0.84 to 0.94)	**38 fewer per 100.000** (from 55 fewer to 21 fewer)	⨁⨁⨁◯ Moderate	CRITICAL
**Cancer Colorrectal Mortality (follow-up: range 9 years; assessed with: FIT)**												
5	non-randomized studies	very serious^b^	not serious	not serious	not serious	none	4545/3,922,028 (0.1%)	18,027/3,513,928 (0.5%)	**RR 0.21** (0.20 to 0.21)	**405 fewer per 100.000** (from 410 fewer to 405 fewer)	⨁⨁◯◯ Low	CRITICAL

*CI*: confidence interval; *RR*: risk ratio. Moderate = the true effect is likely close to the estimate but may be substantially different. Low = the true effect may be substantially different from the estimate

Explanations

a. All studies were classified as some concerns on deviations from intended interventions

b. The main concerns were residual confounding, self-selection bias, and contamination between comparison groups

**Fig. 4:**
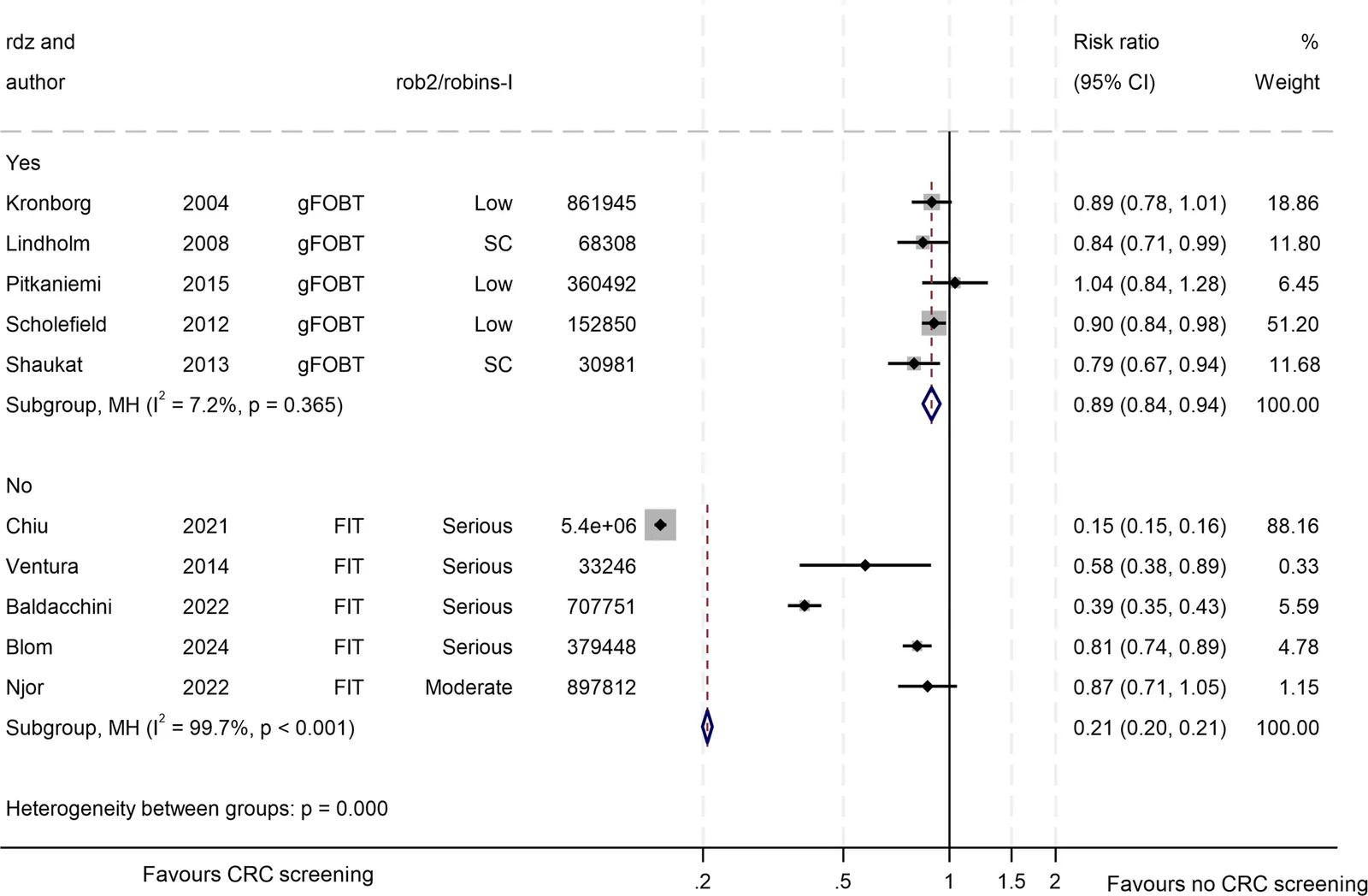
Forest plot showing the effect of colorectal cancer (CRC) screening on CRC-specific mortality compared with no screening. Low = low risk of bias; SC = some concerns, according to the Cochrane Risk of Bias 2 (RoB 2) tool. Results from the random-effects model, as well as the leave-one-out sensitivity analyses, are presented in the Supplementary Material. Note: Weights and between-subgroup heterogeneity test are from Mantel-Haenszel model

In terms of CRC mortality, RCTs evaluating gFOBT reported a pooled RR of 0.89 (95% CI: 0.84–0.94; 5 studies, 1,474,576 participants; moderate-certainty evidence), corresponding to 38 fewer CRC deaths per 100,000 individuals screened (95% CI: 55 fewer to 21 fewer; Fig. 4; Table 2). Leave-one-out sensitivity analyses of the RCTs showed that no single study materially influenced the pooled effect estimate, Supplementary Material. Consistent with these findings, sensitivity analyses using fixed- and random-effects models yielded similar results, Supplementary Material. 

In contrast, among the non-RCTs, pooled estimates differed between models because the study by Chiu et al. contributed approximately 73% of the total study population. Given the predominance of this study, the fixed-effect model was considered the most appropriate and was therefore used for the primary analysis. Non-RCTs evaluating FIT screening reported a pooled RR of 0.21 (95% CI: 0.20–0.21; 5 studies; 7,435,956 participants; low-certainty evidence), corresponding to 405 fewer CRC deaths per 100,000 individuals screened (410 fewer to 405 fewer; Fig. 4; Table 2). When random-effects model was applied, the pooled effect estimate was attenuated and no longer statistically significant (RR 0.47, 95% CI 0.20–1.13).

**Fig. 5:**
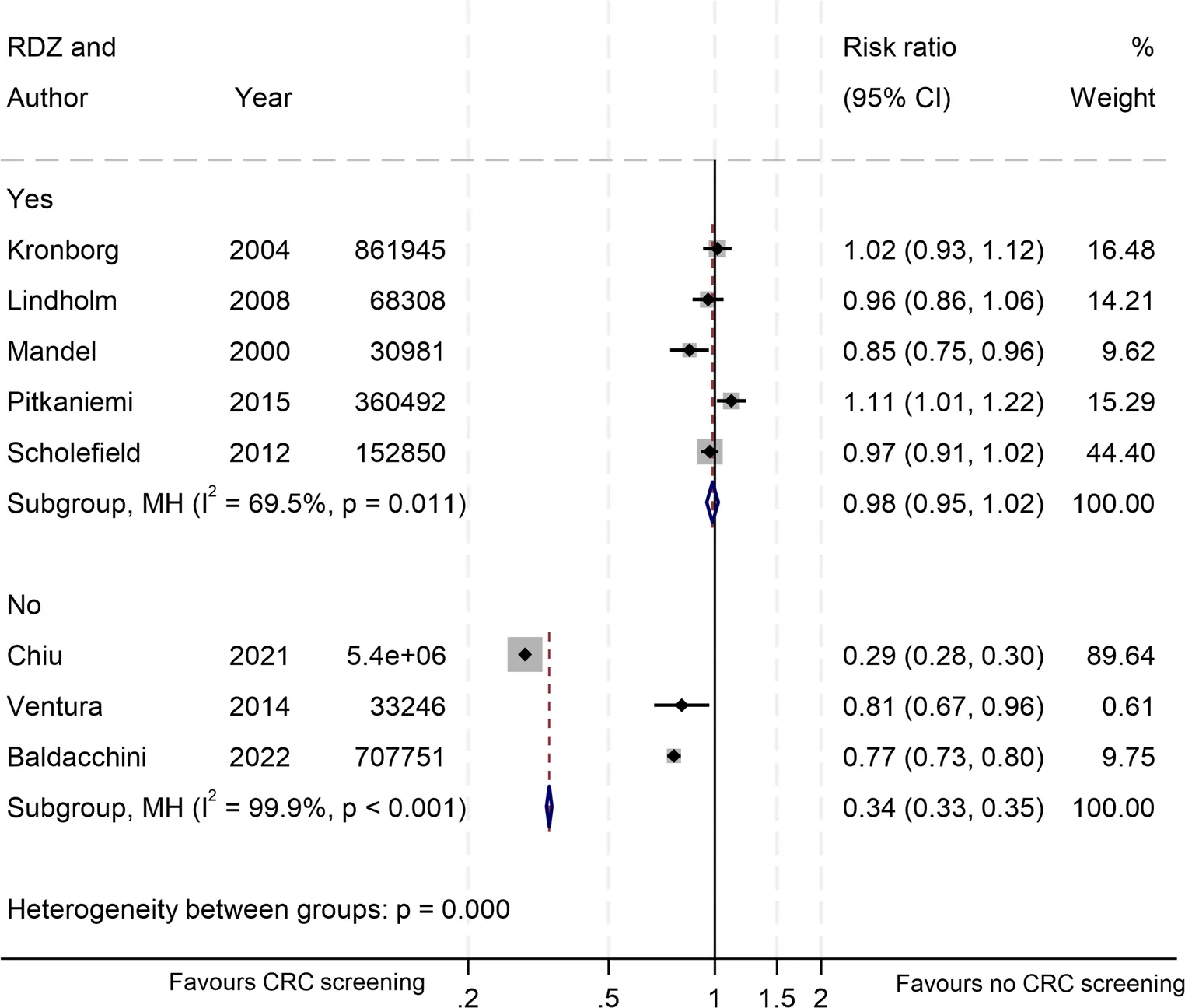
Forest plot of the association between colorectal cancer screening and overall colorectal cancer incidence in screened versus unscreened populations. Note: Weights and between-subgroup heterogeneity test are from Mantel-Haenszel model

For RCTs, CRC screening was not associated with a significant reduction in overall CRC incidence compared with no screening (RR: 0.98, 95% CI: 0.91–1.06; 5 studies, 1,474,576 participants, moderate-certainty evidence Fig. 5). In contrast, among non-RCTs evaluating FIT screening, the primary fixed-effect analysis (largely influenced by the study by Chiu et al., which accounted for approximately 73% of the total study population) demonstrated a substantially lower incidence of CRC among screened individuals (RR: 0.34, 95% CI: 0.33–0.35; 6,158,696 participants).

**Fig. 6:**
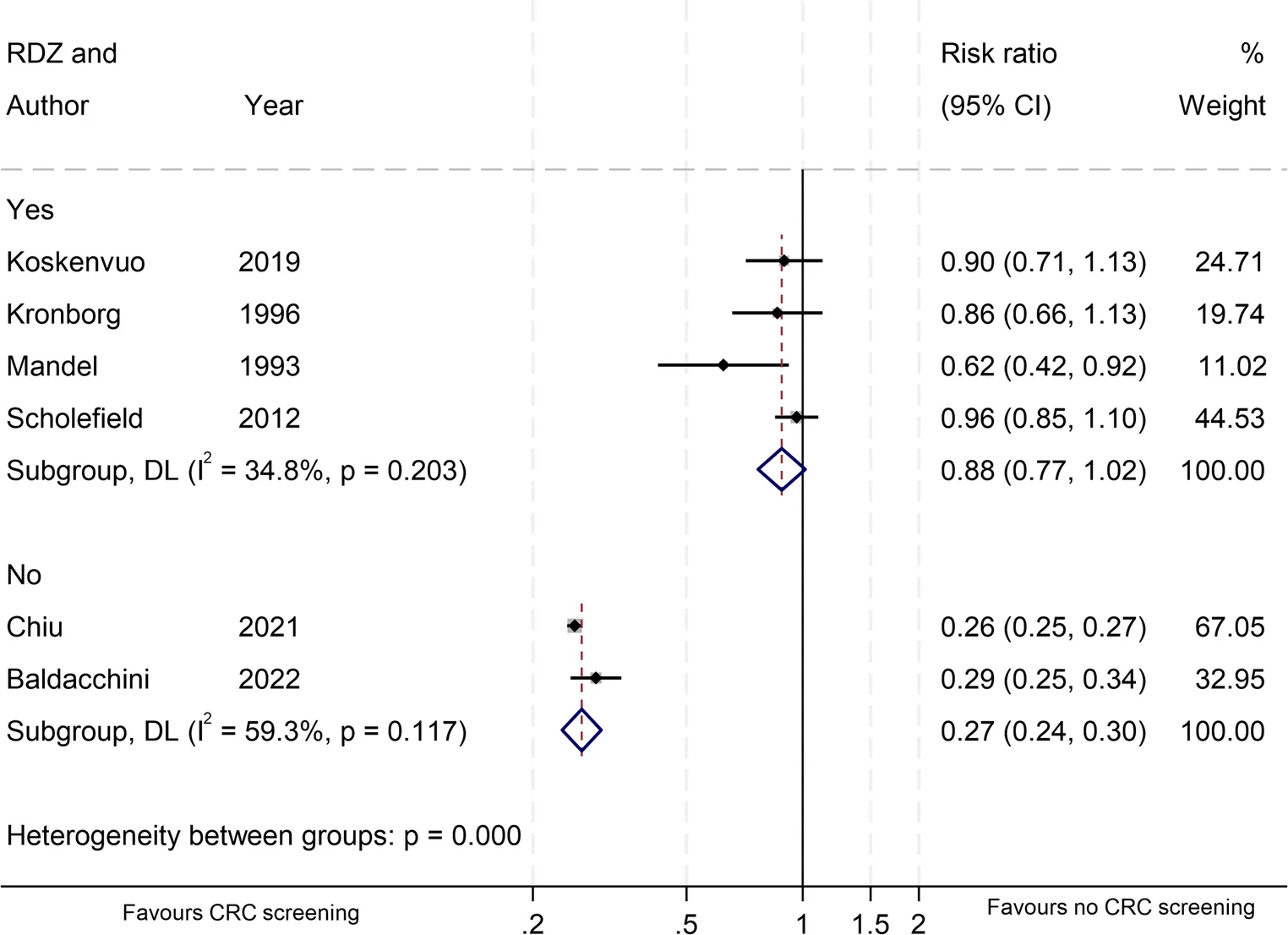
Forest plot of the association between colorectal cancer screening and the incidence of stage IV colorectal cancer, comparing screened and unscreened populations. Note: Weights and between-subgroup heterogeneity test are from random-effects model

Regarding stage-specific outcomes, gFOBT screening evaluated in RCTs was not associated with a significant reduction in the incidence of stage IV CRC compared with no screening (RR: 0.88, 95% CI: 0.77–1.02; 4 studies, 1,367,087 participants; moderate-certainty evidence; Fig. 6). Conversely, non-RCTs evaluating FIT screening showed a markedly lower incidence of stage IV CRC among screened individuals (RR: 0.27, 95% CI: 0.24–0.30; low-certainty evidence; Fig. 6), suggesting a reduction in advanced-stage disease at diagnosis.

Results from the leave-one-out sensitivity analyses and from the alternative meta-analytic models are presented in the Supplementary Material. Specifically, for outcomes primarily analyzed using a fixed-effect model, the corresponding random-effects estimates are provided, whereas for outcomes primarily analyzed using a random-effects model, the corresponding fixed-effect estimates are also reported. 

### Descriptive Results of Included Studies

Regarding safety, only two RCTs reported procedure-related adverse events. In the study by Lindholm et al., four large-bowel perforations occurred following endoscopic polypectomy, corresponding to 0.8% of the 513 adenomas removed [22, 27]. Additionally, one episode of delayed post-polypectomy bleeding was reported 12 days after the procedure, whereas no complications were observed among the 1,987 double-contrast barium enema examinations performed. Long-term follow-up data from the Minnesota Colon Cancer Control Study further suggested that colonoscopy-related complications were uncommon, with an overall complication rate of approximately 0.1%; however, the nature and severity of these adverse events were not reported in detail [25]. Beyond these findings, no additional RCTs or non-RCTs systematically assessed the physical harms associated with CRC screening.

Moreover, several important patient-centered outcomes, including procedure-related complications (such as bleeding and intestinal perforation), quality of life, psychological impact, and the need for subsequent oncological treatments, including chemotherapy or radiotherapy, were not systematically evaluated or reported in the included studies.

## Discussion

In response to the increasing global burden of CRC, fecal occult blood test–based screening strategies have been widely implemented and extensively evaluated. In the present meta-analysis, stool-based screening was associated with a reduction in CRC-specific mortality, representing the principal demonstrated benefit of this intervention. In RCTs, screening resulted in 38 fewer CRC deaths per 100,000 individuals screened (95% CI, 21 to 55 fewer). Although this absolute effect may appear modest at the individual level, organized screening programs target large populations; consequently, even relatively small absolute reductions in mortality may translate into a substantial number of CRC deaths prevented at the population level.

Non-RCTs evaluating FIT screening also showed a reduction in CRC-specific mortality, with larger effect estimates than those observed in the RCTs. Although the certainty of this evidence was rated as low, primarily because of the observational nature of the studies and the potential for residual confounding and selection bias, the larger effect observed with FIT should not necessarily be attributed entirely to limitations of the observational design. Differences in study design preclude a direct comparison between the magnitude of the effects observed with FIT and gFOBT, and some degree of overestimation cannot be excluded. However, it is also plausible that at least part of the observed difference reflects characteristics of FIT itself and of contemporary screening programs.

This interpretation is supported by biological and clinical plausibility. FIT specifically detects human hemoglobin, generally requires only a single stool sample, and does not require dietary restrictions, characteristics that may facilitate participation and adherence to repeated screening [5]. Thus, although the randomized evidence from gFOBT trials cannot be directly extrapolated to FIT, it provides important evidence that repeated stool-based screening can reduce CRC mortality, while the more recent observational studies provide complementary evidence specifically addressing FIT. Taken together, the randomized gFOBT evidence and observational FIT evidence should be regarded as complementary rather than interchangeable, with the latter providing contemporary evidence of FIT effectiveness that is consistent with, and biologically plausible in the context of, the mortality benefit established by randomized gFOBT trials.

Retaining the randomized gFOBT evidence is also particularly important from a public health and policy perspective. Several countries, including Brazil, have not yet implemented organized population-based CRC screening programs, and decisions regarding their introduction depend, in part, on the certainty of evidence for clinically relevant benefits. The randomized gFOBT trials provide moderate-certainty evidence of a reduction in CRC mortality and therefore constitute an important evidence base supporting stool-based screening. If the synthesis were restricted to FIT, evidence for CRC mortality would rely predominantly on non-randomized studies and would consequently be of low certainty, potentially limiting the evidence available to support implementation decisions. Thus, retaining gFOBT trials alongside contemporary FIT studies provides a more comprehensive evidence base for countries considering the implementation of population-based stool-based screening programs.

Additional evidence supporting the association between FIT screening and reduced CRC mortality comes from a recent nested case-control study by Doubeni et al. (2024) [28]. In a large screening-eligible population, completion of at least one FIT within the preceding 5 years was associated with a 33% lower odds of CRC death (adjusted OR, 0.67; 95% CI, 0.59–0.76). Although this study was not eligible for inclusion in our meta-analysis because of its case-control design, its findings provide complementary contemporary evidence supporting the effectiveness of FIT-based screening.

Notably, RCTs did not demonstrate a significant reduction in overall CRC incidence or in the incidence of stage IV CRC at diagnosis. In contrast, non-RCTs evaluating FIT screening showed reductions in both overall CRC incidence and stage IV CRC incidence, suggesting that FIT-based screening may contribute to earlier detection and prevention of advanced disease. Nevertheless, these findings should be interpreted cautiously given the lower certainty of evidence associated with the non-randomized data.

No significant reduction in all-cause mortality was observed, suggesting that the benefits of CRC screening are primarily disease-specific and may not be sufficient to influence overall survival. However, this finding should be interpreted in light of the fact that the randomized evidence was derived predominantly from studies evaluating gFOBT. Compared with FIT, gFOBT has lower sensitivity for the detection of advanced adenomas and colorectal cancer, lower specificity, and more demanding sample handling requirements [29, 30]. Furthermore, gFOBT typically requires dietary restrictions and the collection of multiple stool samples, factors that may adversely affect screening adherence. In contrast, FIT employs antibodies specific to human hemoglobin, does not require dietary restrictions, generally requires fewer samples, and has demonstrated higher sensitivity and participation rates in population-based screening programs. Consistent with these characteristics, the non-RCTs included in our review showed larger reductions in CRC-specific mortality and significant reductions in both overall CRC incidence and stage IV CRC incidence among individuals undergoing FIT screening. Nevertheless, the certainty of this evidence was low because of the inherent limitations of observational studies. Therefore, although FIT-based screening may provide greater benefits through improved participation and more accurate detection of clinically relevant lesions, confirmation from randomized trials evaluating contemporary FIT strategies remains warranted.

Most studies included in our review evaluated biennial screening strategies. Although the Minnesota Colon Cancer Control Study reported numerically greater reductions in CRC mortality with annual than with biennial gFOBT screening, the trial was not designed to directly compare screening intervals, and no statistically significant superiority of annual screening was demonstrated [25]. Importantly, both annual and biennial screening strategies were associated with significant reductions in CRC mortality compared with no screening, supporting the effectiveness of biennial screening programs.

Another relevant aspect concerns the definition of the FIT test cutoff for considering a positive result. There is no consensus on the ideal value. Common cutoff values range from 10 to 20 µg of hemoglobin per gram of stool, but some countries use higher values to reduce the number of colonoscopies needed, even if this implies a slight reduction in the test’s sensitivity. A lower cutoff point increases lesion detection but also generates more false positives, overloading the healthcare system. For CRC, the optimal threshold for a positive result might be between 10 and 20 µg/g (with a false-positive rate of 7%) or 20 µg/g or greater (with a false-positive rate of 5%), with the former increasing colonoscopy resources for false-positive results alone by 40% [31]. The choice of cutoff point represents a balance between sensitivity, specificity, and operational capacity. International guidelines, such as the European Guidelines for Quality Assurance in Colorectal Cancer Screening, recognize this variation and recommend that the cutoff point be adjusted flexibly according to the local context [32]. Recent studies have explored personalizing the cutoff point based on individual factors such as age, sex, and clinical history, which may further improve screening effectiveness [33].

Our findings are consistent with previous systematic reviews demonstrating that fecal occult blood test–based CRC screening reduces CRC-specific mortality. Earlier reviews, including those by Towler et al. and Hewitson et al., established the mortality benefit of stool-based screening based predominantly on randomized trials of gFOBT [34]. Subsequent systematic reviews and meta-analyses have reinforced these findings [3, 35–37]. However, the evidence synthesized in these reviews was largely derived from gFOBT trials, whereas FIT, which is currently preferred in many screening programs, was either not evaluated or was less comprehensively represented. Moreover, several earlier reviews preceded the availability of longer-term follow-up from major screening studies. Importantly, these previous systematic reviews did not formally assess the certainty of the evidence using a structured framework such as GRADE. This is particularly relevant for population-based screening, as the certainty of evidence regarding benefits and harms is an essential consideration for policymakers and health systems when deciding whether to implement organized CRC screening programs, especially in countries where such programs have not yet been established.

Our review further extends the existing literature by evaluating CRC incidence, advanced-stage disease, and safety outcomes, while formally assessing the certainty of evidence for each outcome using the GRADE framework. By integrating randomized evidence from gFOBT trials with contemporary evidence on FIT effectiveness and explicitly evaluating the certainty of the evidence, our findings provide a broader and more clinically relevant evidence base to support decision-making on the implementation of population-based CRC screening programs.

### Limitations and Future Research

This review has several limitations. First, the randomized evidence was derived almost gFOBT, a screening modality that has largely been replaced by FIT in contemporary screening programs because of its superior diagnostic performance and higher participation rates. Consequently, the benefits observed in RCTs may not fully reflect the effectiveness of current FIT-based screening strategies.

Second, although non-RCTs evaluating FIT demonstrated larger reductions in CRC mortality, overall CRC incidence, and stage IV CRC incidence, the certainty of this evidence was low because of the inherent limitations of observational study designs, including the potential for residual confounding, selection bias, and health-user effects. Therefore, causal inferences regarding the magnitude of benefit associated with FIT screening should be made cautiously.

Third, important patient-centered outcomes were infrequently reported. Data on screening-related harms, including colonoscopy-associated bleeding and perforation, psychological effects, quality of life, overdiagnosis, and the need for subsequent oncological treatments were limited or unavailable. As a result, the balance between benefits and harms could not be comprehensively assessed.

Finally, substantial variation existed across studies with respect to screening intervals, follow-up duration, participation rates, and diagnostic pathways following a positive screening test. These differences may have contributed to variability in effect estimates and limit the direct comparability of results across screening programs.

Future research should prioritize RCTs evaluating contemporary FIT-based screening strategies, including different FIT thresholds and screening intervals. Studies should also incorporate patient-centered outcomes, adverse events, quality-of-life measures, and cost-effectiveness analyses to better inform screening policies. In addition, long-term comparative studies are needed to determine the optimal FIT screening frequency and to evaluate whether the larger benefits observed in observational studies can be confirmed in randomized settings.

## Conclusion

In conclusion, this systematic review demonstrated that CRC screening, although not associated with a reduction in overall mortality, significantly reduces CRC mortality. Observational studies suggest that FIT-based screening may provide additional advantages, including reductions in CRC incidence and advanced-stage disease at diagnosis.

These findings indicate that screening is effective for early detection, which may allow for earlier interventions and, consequently, better outcomes related to the disease. In view of these findings, decisions about the implementation and recommendation of population-based screening programs should be considered. Studies are also needed on aspects such as cost-effectiveness, patient preferences, burden of examinations, and risks associated with screening.

## Supplementary Information

Below is the link to the electronic supplementary material.

Supplementary Material 1 (DOCX 145 KB)

## Data Availability

No datasets were generated or analysed during the current study.
