# Supplementary material for: Effectiveness and Safety of Colorectal Cancer Screening Via Fecal Occult Blood Testing: A Systematic Review and Meta-Analysis
Source: J Gastrointest Cancer. 2026 Sep 23;57(1):223. doi: 10.1007/s12029-026-01605-9 (PMC13601071; doi:10.1007/s12029-026-01605-9)
Supplement: Supplementary file 1 [file 12029_2026_1605_MOESM1_ESM.docx]

**Effectiveness and Safety of Colorectal Cancer Screening via Fecal Occult Blood Testing: A Systematic Review and Meta-Analysis**

**Mayra Calil Jorge Frankenfeld¹; Giovanna Camarotto Patah²; Luiza Teixeira Soares¹; Sarah Nascimento Silva³; Fernando Gomes Romeiro¹; Vania dos Santos Nunes Nogueira¹**

1. Universidade Estadual Paulista (UNESP), Faculdade de Medicina, Botucatu, Sao Paulo, Brazil
2. Faculty of Medicine of Jundiaí (FMJ), Jundiaí, São Paulo, Brazil
3. René Rachou Institute, Oswaldo Cruz Foundation, FIOCRUZ, Belo Horizonte, Minas Gerais, Brazil

* Correspondence:

Vania dos Santos Nunes Nogueira

E-mail: [vania.nunes-nogueira@unesp.br](mailto:vania.nunes-nogueira@unesp.br)

**Summary**

[**Table S1**. Excluded Studies and Reasons for Exclusion	3](#_Toc231812575)

[References of the excluded studies	4](#_Toc231812576)

[**Table S2**. Risk of bias of the non-randomized controlled trials according to ROBINS-I tool	7](#_Toc231812577)

[Supplementary Figures	8](#_Toc231812578)

[**Figure S1.** Forest plot of all-cause mortality comparing colorectal cancer (CRC) screening and control groups using a fixed-effect Mantel–Haenszel model.	8](#_Toc231812579)

[**Figure S2.** Forest plot of colorectal cancer (CRC) mortality comparing CRC screening and control groups using a random-effects model	9](#_Toc231812580)

[**Figure S3.** Forest plot of overall colorectal cancer incidence comparing screened and unscreened populations using a random-effects model.	10](#_Toc231812581)

[**Figure S4.** Leave-one-out sensitivity analysis of the effect of colorectal cancer screening on general mortality in randomized controlled trials using a random-effects model.	11](#_Toc231812582)

[**Figure S5.** Leave-one-out sensitivity analysis of the effect of colorectal cancer screening on CRC-specific mortality in non-randomized studies using a fixed-effect model.	12](#_Toc231812583)

[**Figure S6**. Leave-one-out sensitivity analysis of the effect of colorectal cancer screening on CRC-specific mortality in non-randomized studies using a random-effects model.	13](#_Toc231812584)

[**Figure S7.** Forest plot of stage IV colorectal cancer incidence comparing screened and unscreened populations using a fixed-effect model.	14](#_Toc231812585)

[Search strategies for MEDLINE, EMBASE, CENTRAL, and LILACS.	15](#_Toc231812586)

# **Table S1**. Excluded Studies and Reasons for Exclusion

| **Study** | **Reasons for exclusion** |
| --- | --- |
| **FAIVRE 2004** | It was a non-randomized gFOBT screening study (1) |
| **Forsberg 2022** | The study did not report either overall or colorectal cancer mortality (2) |
| **Hardcastle 1996** | Overlapping data with another included study (3) |
| **Ibanez-Sanz 2021** | It is a review and does not include primary data (4) |
| **Jorgensen 2002** | Overlapping data with another included study (5) |
| **Kaalby 2022** | Overlapping data with another included study (6) |
| **Klaaborg 1986** | Overlapping data with another included study.(7) |
| **Koskenvuo 2019** | Overlapping data with another included study (8) |
| **Kronborg, 1987** | Overlapping data with another included study (9) |
| **Kronborg, 1994** | Overlapping data with another included study (10) |
| **Kronborg, 1996** | Overlapping data with another included study (11) |
| **Larsen 2018** | The study did not report either overall or colorectal cancer mortality (12) |
| **Lee 2007** | It was a non-randomized gFOBT screening study (13) |
| **Libby 2012** | It was a non-randomized gFOBT screening study (14) |
| **Luu 2022** | It was a case-control study (15) |
| **Malila 2007** | It was a non-randomized gFOBT screening study (16) |
| **Mandel 1999** | Overlapping data with another included study (17) |
| **Paimela 2020** | Overlapping data with another included study (18) |
| **Robinson 1999** | Adverse events were not reported by study group (19) |
| **Scholefield 2002** | Overlapping data with another included study (20) |
| **Steffen 2014** | The study did not evaluate population-based colorectal cancer screening (21) |
| **Sung 2022** | It was a case-control study (22) |
| **Whynes, 2010** | Overlapping data with another included study (23) |

# References of the excluded studies

1.	Faivre J, Dancourt V, Lejeune C, Tazi MA, Lamour J, Gerard D, et al. Reduction in colorectal cancer mortality by fecal occult blood screening in a French controlled study. Gastroenterology. 2004;126(7):1674-80.

2.	Forsberg A, Westerberg M, Metcalfe C, Steele R, Blom J, Engstrand L, et al. Once-only colonoscopy or two rounds of faecal immunochemical testing 2 years apart for colorectal cancer screening (SCREESCO): preliminary report of a randomised controlled trial. Lancet Gastroenterol Hepatol. 2022;7(6):513-21.

3.	Hardcastle JD, Chamberlain JO, Robinson MH, Moss SM, Amar SS, Balfour TW, et al. Randomised controlled trial of faecal-occult-blood screening for colorectal cancer. Lancet. 1996;348(9040):1472-7.

4.	Ibáñez-Sanz G, Sanz-Pamplona R, Garcia M, On Behalf Of The Msic-Sc Research G. Future Prospects of Colorectal Cancer Screening: Characterizing Interval Cancers. Cancers (Basel). 2021;13(6).

5.	Jørgensen OD, Kronborg O, Fenger C. A randomised study of screening for colorectal cancer using faecal occult blood testing: results after 13 years and seven biennial screening rounds. Gut. 2002;50(1):29-32.

6.	Kaalby L, Al-Najami I, Deding U, Berg-Beckhoff G, Steele RJC, Kobaek-Larsen M, et al. Cause of Death, Mortality and Occult Blood in Colorectal Cancer Screening. Cancers (Basel). 2022;14(1).

7.	Klaaborg K, Madsen MS, Søndergaard O, Kronborg O. Participation in mass screening for colorectal cancer with fecal occult blood test. Scand J Gastroenterol. 1986;21(10):1180-4.

8.	Koskenvuo L, Malila N, Pitkäniemi J, Miettinen J, Heikkinen S, Sallinen V. Sex differences in faecal occult blood test screening for colorectal cancer. British Journal of Surgery. 2019;106(4):436-47.

9.	Kronborg O, Fenger C, Søndergaard O, Pedersen KM, Olsen J. Initial mass screening for colorectal cancer with fecal occult blood test. A prospective randomized study at Funen in Denmark. Scand J Gastroenterol. 1987;22(6):677-86.

10.	Kronborg O, Wahrendorf J. Colorectal cancer screening: methods, benefits and costs. Eur J Cancer. 1994;30a(6):877-9.

11.	Kronborg O, Fenger C, Olsen J, Jørgensen OD, Søndergaard O. Randomised study of screening for colorectal cancer with faecal-occult-blood test. Lancet. 1996;348(9040):1467-71.

12.	Larsen MB, Njor S, Ingeholm P, Andersen B. Effectiveness of Colorectal Cancer Screening in Detecting Earlier-Stage Disease-A Nationwide Cohort Study in Denmark. Gastroenterology. 2018;155(1):99-106.

13.	Lee KJ, Inoue M, Otani T, Iwasaki M, Sasazuki S, Tsugane S. Colorectal cancer screening using fecal occult blood test and subsequent risk of colorectal cancer: a prospective cohort study in Japan. Cancer Detect Prev. 2007;31(1):3-11.

14.	Libby G, Brewster DH, McClements PL, Carey FA, Black RJ, Birrell J, et al. The impact of population-based faecal occult blood test screening on colorectal cancer mortality: a matched cohort study. Br J Cancer. 2012;107(2):255-9.

15.	Luu XQ, Lee K, Jun JK, Suh M, Jung KW, Choi KS. Effect of colorectal cancer screening on long-term survival of colorectal cancer patients: Results of the Korea National Cancer Screening Program. Int J Cancer. 2022;150(12):1958-67.

16.	Malila N, Hakama M, Pukkala E. A 25-year follow-up of a population screened with faecal occult blood test in Finland. Acta Oncol. 2007;46(8):1103-6.

17.	Mandel JS, Church TR, Ederer F, Bond JH. Colorectal cancer mortality: effectiveness of biennial screening for fecal occult blood. J Natl Cancer Inst. 1999;91(5):434-7.

18.	Paimela H, Malila N, Palva T, Hakulinen T, Vertio H, Järvinen H. Early detection of colorectal cancer with faecal occult blood test screening. Br J Surg. 2010;97(10):1567-71.

19.	Robinson MH, Hardcastle JD, Moss SM, Amar SS, Chamberlain JO, Armitage NC, et al. The risks of screening: data from the Nottingham randomised controlled trial of faecal occult blood screening for colorectal cancer. Gut. 1999;45(4):588-92.

20.	Scholefield JH, Moss S, Sufi F, Mangham CM, Hardcastle JD. Effect of faecal occult blood screening on mortality from colorectal cancer: results from a randomised controlled trial. Gut. 2002;50(6):840-4.

21.	Steffen A, Weber MF, Roder DM, Banks E. Colorectal cancer screening and subsequent incidence of colorectal cancer: results from the 45 and Up Study. Med J Aust. 2014;201(9):523-7.

22.	Sung SY, Choi HH, Kim S, Park BR, Kim YK, Kim HK, et al. Colonoscopy decreases mortality in colorectal cancer patients compared with fecal immunochemical test. J Gastroenterol Hepatol. 2022;37(10):1991-7.

23.	Whynes DK, Frew EJ, Manghan CM, Scholefield JH, Hardcastle JD. Colorectal cancer, screening and survival: the influence of socio-economic deprivation. Public Health. 2003;117(6):389-95.

# **Table S2**. Risk of bias of the non-randomized controlled trials according to ROBINS-I tool

| ROBINS-I Domain | Blom et al. (2024) | Njor  (2022) | Chiu  (2015) | Ventura  (2014) | Baldacchini  (2022) |
| --- | --- | --- | --- | --- | --- |
| Bias due to confounding | Moderate | Moderate | Serious | Serious | Moderate |
| Bias in selection of participants into the study | Low | Low | Moderate | Moderate | Serious |
| Bias in classification of interventions | Low | Low | Low | Low | Low |
| Bias due to deviations from intended interventions | Serious | Moderate | Moderate | Moderate | Serious |
| Bias due to missing data | Low | Low | Low | Low | Low |
| Bias in measurement of outcomes | Low | Low | Low | Low | Low |
| Bias in selection of the reported result | Low | Low | Low | Low | Low |
| Overall ROBINS-I judgment | Serious | Moderate | Serious | Serious | Serious |

# Supplementary Figures

# **Figure S1.** Forest plot of all-cause mortality comparing colorectal cancer (CRC) screening and control groups using a fixed-effect Mantel–Haenszel model.

# **Figure S2.** Forest plot of colorectal cancer (CRC) mortality comparing CRC screening and control groups using a random-effects model

# **Figure S3.** Forest plot of overall colorectal cancer incidence comparing screened and unscreened populations using a random-effects model.

# **Figure S4.** Leave-one-out sensitivity analysis of the effect of colorectal cancer screening on general mortality in randomized controlled trials using a random-effects model.

Each study was sequentially excluded from the meta-analysis, and the pooled relative risk (RR) was recalculated to assess the influence of individual studies on the overall estimate. The diamond represents the pooled effect estimate from the complete meta-analysis including all studies. The vertical lines represent the pooled effect estimates obtained after omitting each study in turn, along with their corresponding 95% confidence intervals. Minimal changes in the pooled estimates across iterations indicate that no single study exerted a disproportionate influence on the overall result, supporting the robustness of the findings

# **Figure S5.** Leave-one-out sensitivity analysis of the effect of colorectal cancer screening on CRC-specific mortality in non-randomized studies using a fixed-effect model.

# **Figure S6**. Leave-one-out sensitivity analysis of the effect of colorectal cancer screening on CRC-specific mortality in non-randomized studies using a random-effects model.

# **Figure S7.** Forest plot of stage IV colorectal cancer incidence comparing screened and unscreened populations using a fixed-effect model.

# Search strategies for MEDLINE, EMBASE, CENTRAL, and LILACS.

**PubMed**

#1 "Colonic Neoplasms"[Mesh] OR (Colonic Neoplasm) OR (Neoplasm, Colonic) OR (Neoplasms, Colonic) OR (Colon Neoplasms) OR (Colon Neoplasm) OR (Neoplasm, Colon) OR (Neoplasms, Colon) OR (Cancer of Colon) OR (Colon Cancers) OR (Cancer of the Colon) OR (Colonic Cancer) OR (Cancer, Colonic) OR (Cancers, Colonic) OR (Colonic Cancers) OR (Colon Cancer) OR (Cancer, Colon) OR (Cancers, Colon) OR "Rectal Neoplasms"[Mesh] OR (Neoplasm, Rectal) OR (Rectal Neoplasm) OR (Rectum Neoplasms) OR (Neoplasm, Rectum) OR (Rectum Neoplasm) OR (Rectal Tumors) OR (Rectal Tumor) OR (Tumor, Rectal) OR (Neoplasms, Rectal) OR (Cancer of Rectum) OR (Rectum Cancers) OR (Rectal Cancer) OR (Cancer, Rectal) OR (Rectal Cancers) OR (Rectum Cancer) OR (Cancer, Rectum) OR (Cancer of the Rectum) OR (Colorectal Cancer) OR (Rectal Cancer)

**AND**

#2 ("Occult Blood"[Mesh] OR (occult blood test) OR (fecal immunochemical test) OR (faecal occult blood test) OR (FOB test) OR (FIT test) OR (Hemoccult Test) OR (guaiac-based fecal occult blood testing) OR (gFOBT))

**AND**

#3 (((randomized controlled trial[pt] OR controlled clinical trial[pt] OR randomized[tiab] OR placebo[tiab] OR clinical trials as topic[mesh:noexp] OR randomly[tiab] OR trial[ti] NOT (animals[mh] NOT humans [mh])))) OR"Cohort Studies"[Mesh] OR (Cohort Study) OR (Studies, Cohort) OR (Study, Cohort)

**Embase**

#1 'occult blood test'/exp OR 'fecal immunochemical test' OR 'occult blood test kit'/exp OR 'faecal occult blood test kit' OR 'fecal occult blood test kit' OR 'FlexSure OBT' OR 'FOB test kit' OR 'Hemastix' OR 'HemeSelect' OR 'Hemo Techt NS-Plus' OR 'Hemoccult ICT' OR 'Hemoccult II' OR 'InSure FIT' OR 'MagStream HemSp'

#2 'colorectal cancer'/exp OR 'colorectal carcinoma'

**AND**

#3 'crossover procedure':de OR 'double-blind procedure':de OR 'randomized controlled trial':de OR  'single-blind procedure':de OR (random* OR  factorial* OR crossover* OR cross NEXT/1 over* OR placebo* OR doubl* NEAR/1 blind* OR singl* NEAR/1 blind* OR assign* OR allocat* OR volunteer*):de,ab,ti

**Lilacs (Bireme)**

#1 MH:"Neoplasias colorretais" OR (Colonic neoplasm) OR (Colon neoplasm) OR (Cancer of colon) OR (Colon cancer) OR (Rectal neoplasm) OR (Rectal cancer) or (Rectal tumor) OR (Cancer of rectum) OR (Colorectal cancer) OR MH:C04.588.274.476.411.307$ OR MH:C06.301.371.411.307$ OR MH:C06.405.249.411.307$ OR MH:C06.405.469.158.356$ OR MH:C06.405.469.491.307$ OR MH:C06.405.469.860.180$

**AND**

#2 MH:"Sangue oculto" OR (Occult blood) OR (Occult blood test) OR (fecal immunochemical test) OR (faecal occult blood test) OR (FOB test) OR (FIT test) OR (Hemoccult test) OR (guaiac-based fecal occult blood testing) OR (gFOBT) OR MH:E01.370.225.925$ OR MH:E05.200.925$

**Cochrane Library**

#1 Colonic neoplasms

#2 Rectal neoplasms

#3 Colerectal neoplasms

#4 Occult blood

#5 (occult blood test) OR (fecal immunochemical test) OR (faecal occult blood test) OR (FOB test) OR (FIT test) OR (Hemoccult Test) OR (guaiac-based fecal occult blood testing) OR (gFOBT)

#6 Mass Screening
